# Journal of Biomedical Science, marking a new epoch: moving to open access in 2009

**DOI:** 10.1186/1423-0127-16-1

**Published:** 2009-01-08

**Authors:** Michael MC Lai

**Affiliations:** 1 National Cheng Kung University, Tainan City 701, Taiwan

Welcome to the new *Journal of Biomedical Science *(JBS), which is published by BioMed Central in partnership with the National Science Council, Taiwan (NSC). This volume marks the transition of the journal from a subscription journal to an "open access" journal. Starting from January 2009, volume 16, the journal will be published only in the electronic format and freely accessible online at [1].

With the development of the internet, the way to conduct and share scientific research has been changing dramatically. Though still challenged by commercial journal publishers, open access publishing has become a growing trend for the possibility of expanding the circulation of scientific work and maximizing the research. Accumulating evidence has shown that open access articles are cited more quickly and more frequently than non-open access article published in the same journal [2-4]. That probably explains the increasing interest from traditional journals in adopting open access as their new way of publishing [5].

Sponsored by the NSC, JBS aims to serve as an international forum for encouraging interdisciplinary discussions and contributing to the advancement of biomedical science, rather than as a periodical for commercial gain. During the past years, the NSC had partnered with established publishers to publish the journal under the traditional subscription model. The visibility and impact of the journal has risen steadily but still has considerable room for improvement. We are well aware that access to an article, by itself, is not sufficient for citation. But access to it, by any path, is still a necessary pre-condition for citation [6]. More and more journals are now turning to the open access publishing model [7]. Therefore, to accelerate the dissemination of research information and provide maximum access to scholarly communication, the journal's Editors and the NSC have decided to adopt the open access model and have chosen BioMed Central as our partner for the transition to open access. We believe that the sooner we make this transition, the sooner JBS will reach more readers and authors around the world.

By taking advantage of the new open access model, we will be able to publish the journal in a more timely and readable fashion. All the accepted articles starting from volume 16 will be published immediately upon acceptance with the attractive features of unlimited space for figures, extensive datasets and video footage. Open access articles are accessible online in full text to all interested readers or libraries without financial or permission barriers. Previous articles published before 2009 are also freely available online (in final PDF version) to readers in the journal's local repository [8].

We realize that the quality of a journal depends mainly on the quality of the articles it publishes. We welcome manuscripts reporting results from original research on fundamental and molecular aspects of basic medical sciences. Review articles of current interests are highly desired. Undeniably, open access also raises the issue of increasing financial burdens on authors because the publication cost is traditionally borne by the authors. Thanks to support from the NSC, authors will not be required to pay any article processing charges to publish their scientific works in JBS.

Following some initial difficulties during the transition from the old to the new system, the online submission and review process are now running smoothly. I anticipate that we will have a very smooth publication in the new format from now on. I would like to attribute the credit for this achievement to our members of the international Editorial Board, the staffs at the NSC and BioMed Central.

Growing in quality and recognition, JBS is indexed in more than 10 prominent scientific databases, including the Science Citation Index (SCI), MEDLINE, EMBASE, BIOSIS, CA SEARCH, AIDSLINE, TOXLINE, Life Sciences Collection, etc. With the continuous support of scientists from more than 30 countries, it has been able to attract quality articles from authors spanning all the continents. We believe that this new format and innovative approach to publishing will facilitate JBS as an indispensible source of biomedical science knowledge everywhere.

We sincerely hope that you continue to access this new-look journal, to enjoy the selection of articles and features, to submit manuscripts for publication, and to share your valuable thoughts regarding the contents of the journal.
